# Periodontal Evaluation for a New Alkasite Restorative Material in Noncarious Cervical Lesions: A Randomized‐Controlled Clinical Trial

**DOI:** 10.1002/cre2.70025

**Published:** 2024-10-13

**Authors:** Khattab Mustafa, Ghaith Alfakhry, Hussam Milly

**Affiliations:** ^1^ Faculty of Philosophy and Social Sciences University of Augsburg Augsburg Germany; ^2^ Faculty of Dental Medicine Damascus University Damascus Syria; ^3^ Department of Education University of Oxford Oxford UK

**Keywords:** dental materials, dental plaque index, gingival bleeding on probing, gingival retraction techniques

## Abstract

**Objectives:**

This study aims to evaluate the periodontal condition adjacent to Cention N (CN) restorations applied for noncarious cervical lesions (NCCL) compared with resin‐modified glass ionomer cement (RM‐GIC) restorations in terms of plaque accumulation, attachment loss, and gingival inflammation.

**Materials and Methods:**

This is a double‐blind split‐mouth three‐armed randomized‐controlled clinical trial. The study arms are RM‐GIC (FUJI II LC), CN + adhesive system, and CN + retentive groove. The study included 25 restorations per arm. Follow‐ups were performed at 1 week, 3, 6, and 9 months after the application of the restorations. The periodontal condition was evaluated using the plaque index (PI), the bleeding on probing index (BOP), and the probing depth (PD). Appropriate tests were used to perform statistical analyses (*α* = 0.05).

**Results:**

There was no significant difference between Cention N and RM‐GIC regarding the studied variables. However, after the application of the restorations, it was noted that the PI and the PD mean values increased. The average increase after 9 months was 0.06 and 0.34 for PI and PD, respectively, with that of PD being significant. It was also noted that the percentage of positive BOP sites increased at the 1‐week follow‐up for all study groups and afterward dropped to near preintervention values at the 3‐month and later follow‐ups.

**Conclusions:**

The two ion‐releasing materials Cention N and RM‐GIC have a comparable and clinically acceptable effect on the gingival tissue when restoring NCCLs. The combined effect of the gingival retraction cord and the rubber dam clamp on the periodontal tissues might be more important to consider, especially in relation to the probing depth. Future long‐term studies are needed to evaluate the effect of Cention N on the subgingival biofilm in comparison with nonion‐releasing restorative materials, and subsequently, its effect on gingival inflammation.

**Clinical Trial Registration:**

This clinical trial was registered in clinicaltrial.gov clinical registry under protocol #NCT05593159.

## 1 Introduction

Noncarious cervical lesions (NCCLs) arise as a result of the loss of a tooth's hard tissues from the cervical region through processes unrelated to the biofilm. About 46% of adults have NCCLs, whereas their prevalence increases in older populations (Teixeira et al. 2020). The genesis of NCCLs could be attributed to several factors. Predominantly, these factors include abrasive toothbrushing practices and consumption of acidic dietary substances; the effect of occlusal factors is nonetheless not conclusive (Goodacre, Eugene Roberts, and Munoz 2023).

Treatment of NCCLs is indicated for esthetic and hypersensitivity‐related reasons. A restorative, surgical, or combined approach could be considered. The treatment plan for NCCLs should also target their etiologies to interrupt lesions' development (Goodacre, Eugene Roberts, and Munoz 2023). To restore NCCLs, glass ionomer cement (GIC) and resin composite (RC) are commonly used. A recent meta‐analysis showed no significant difference between the two materials regarding all the following parameters: marginal discoloration, marginal adaptation, secondary caries, color, anatomic form, and surface texture. In terms of the retention rate, GIC showed significantly better performance (Bezerra et al. 2020).

In terms of the gingival‐related parameters adjacent to dental materials, these clinical outcomes have not been studied well in the long term. Nevertheless, some studies found that different restorative materials have different effects on the subgingival biofilm; a case in point is that amalgam and GIC have a better effect on the combination of a subgingival biofilm when compared with RC. Despite this, the clinical manifestations of the phenomenon are still not clear. (Paolantonio et al. 2004; Santos et al. 2007). One cross‐sectional study found that NCCLs restored with RC had a significantly higher percentage of bleeding sites compared with nonrestored NCCLs, which could be attributed to the increase in plaque accumulation (Gurgel et al. 2016).

Cention N (CN) (Ivoclar‐Vivadent, Liechtenstein) is a relatively new alkasite restorative material and it is the first available bioactive RC (Tiskaya et al. 2019). The liquid of CN consists of four different monomers, chemo polymerization, and photopolymerization activators. It does not contain any acidic monomers or water. The powder consists of reactive and nonreactive fillers such as barium aluminum silicate glass, ytterbium trifluoride, iso filler, calcium barium aluminum fluorosilicate glass, and calcium fluorosilicate glass (Tiskaya et al. 2019). The calcium ion release of Cention N was found to be the highest among 13 other restorative materials, and the fluoride and hydroxyl ion release was found to be acceptable (Ruengrungsom et al. 2020). Although Cention N is classified as RC, its ion release property may have a positive impact on the subgingival biofilm and may subsequently show a better gingival response compared with traditional RC, as this property is believed to be associated with an increase in the biofilm pH degree and an antibacterial effect (Daabash et al. 2023; Wiriyasatiankun, Sakoolnamarka, and Thanyasrisung 2022). The mechanical and ion‐releasing properties of this material have been evaluated in vitro (Tiskaya et al. 2019). However, only a few randomized‐controlled trials of Cention N are available, whereas, to the best of our knowledge, the gingival‐related clinical performance of Cention N in NCCLs has not been reported in the dental literature.

According to the manufacturer's instructions, one of Cention N's indications is NCCLs. These lesions could often be in contact with compromised gingival tissue due to gingival recession (Naik, Jacob, and Nainar 2016). Studying Cention N's gingival‐related clinical outcomes could provide clinicians with information about its performance, especially in such areas, and shed light on the potential benefits of ion‐releasing materials for gingival health.

The aim of this clinical trial is to evaluate the periodontal response to Cention N restorations applied with or without an adhesive system in comparison with the standard restorative material for NCCLs: RM‐GIC (Bezerra et al. 2020). The null hypothesis tested was that Cention N with or without an adhesive system yields the same periodontal response as RM‐GIC.

## 2 Materials and Methods

### 2.1 Protocol Registration, and Ethics Approval

This study design followed the Consolidated Standards of Reporting Trials (CONSORT) statement (Schulz, Altman, and Moher 2010). Ethical approval was obtained from Damascus University no./2777/2021. Written informed consent was obtained from all participants before starting the restorative procedures after a thorough explanation of the study aims, procedures, risks, and benefits was provided. The study design of this trial was registered at clinicaltrials.org, NCT05593159.

### 2.2 Study Design, and Sittings

This trial is a double‐blind, split‐mouth randomized‐controlled trial. Clinical procedures and follow‐ups were conducted at the Department of Restorative Dentistry, Faculty of Dentistry, Damascus University, Syria, during the period from May 2022 to November 2023.

### 2.3 Sample Size and Recruitment

To determine the sufficient sample size for the study, a pilot study with the same design as the present study was conducted. The pilot study included six participants who were evaluated for the retention of restorations after 9 months. PASS software (RRID:SCR_019099) was used for sample size calculation. With an α of 0.05, a power of 90%, and a two‐sided test, utilizing the Paired Wilcoxon Signed‐Rank Test, the minimal sample size was 19 in each group. After including a 20% dropout rate, the sample size per arm was 24. The inclusion criteria for patients were as follows: 18 years of age or older, in good general and periodontal health, with no abnormal tooth mobility or deep pockets, acceptable oral hygiene (teeth brushing at least once a day, and no generalized plaque accumulation), having at least 20 teeth under occlusion, and presence of three or more NCCLs that are deeper than 1 mm and involve both the enamel and dentin of vital teeth. The exclusion criteria were as follows: pregnancy, lactation, active severe bruxism habits, or xerostomia (Loguercio et al. 2018; Santos et al. 2007). Patients were examined by two calibrated qualified restorative dentistry specialists to determine if they fulfilled the inclusion criteria.

### 2.4 Random Sequence Generation and Allocation Concealment

Intra‐individual randomization was carried out using Microsoft Excel for Windows (RRID:SCR_016137); thus, every patient received three restorations randomly, one from each arm of the study. Randomization results were concealed from the operator using sequentially numbered, opaque, sealed envelopes. These envelopes were not opened until the patient's arrival. Restorative dental procedures were performed on allocated lesions starting with the tooth of the lowest number (universal numbering system) and moving clockwise.

### 2.5 Intervention

Information about teeth brushing and oral and dietary habits was obtained to help instruct each patient on how to maintain their oral health and avoid traumatic brushing. At each follow‐up, the patients were reinstructed to ensure their adherence.

Gingival health parameters were measured using a CP15 UNC probe (548/4 Medesy; Maniago, Italy). These parameters were plaque accumulation using the plaque index (PI) introduced by Silness and Loe (Table 1) (Silness and Löe 1964), probing depth (PD) in millimeters, and bleeding on probing (BOP) (yes or no). Both PD and BOP were measured at three points for each tooth (mesiobuccal, mid‐buccal, and distobuccal).

**Table 1:** Plaque index PI.

Grade	Description
0	No plaque
1	A thin plaque layer at the gingival margin that is only detectable by scraping with a probe.
2	A moderate layer of plaque along the gingival margin. Interdental spaces are free of plaque; however, plaque is visible to the naked eye.
3	Abundant plaque along the gingival margin and interdental spaces filled with plaque.

All operative procedures were conducted by one operator. To calibrate the procedures, five restorations per study group were placed following the protocol of this study. These restorations were not included in the study.

Before restorative procedures, local anesthesia was administered using 3% Mepivacaine. Afterward, the tooth surface was cleaned using pumice (ProphPaste Pro‐N100, Switzerland) in a rubber cup under water cooling. Then, a retentive cord (Sure‐cord, Korea) was placed with minimum pressure using Gingival Cord Packer Universal (585, Medesy, Italy) and a rubber dam was placed. HYGENIC dental dam B clamp sets (Coltene/Whaledent, Inc. USA) were used to facilitate retraction of gingival tissue.

Each patient received a single RM‐GIC and two Cention N restorations (Batch No. Z01V4K). In terms of Cention N restorations, the manufacturing company recommends using Cention N (Ivoclar‐Vivadent, Liechtenstein, Switzerland) with a universal adhesive system (UA) in nonretentive cavities or with no adhesive system in retentive cavity preparations. Therefore, before application of restoration, half of the NCCLs planned to be restored by Cention N were pretreated as follows: First, UA (Tetric N‐Bond Universal, Liechtenstein) was applied by a micro‐brush and rubbed against the tooth surface for 20 s. Second, an air spray was gently applied to disperse the adhesive until a glossy, firm layer resulted. Third, the adhesive was light‐cured for ten seconds (1200 mW/cm^2^) using woodpecker curing light LED‐F, China, which was calibrated before each use. The other half was treated as follows: NCCLs' surfaces were roughened gently using a round carbide bur (H1SEM.204.014 VPE5 or H1SEM.204.016 VPE 5, Komet Dental, Lemgo, Germany) on a low‐speed handpiece (Each bur was used for no more than five lesions). Thereafter, a fine gingival retentive groove (RG) was made approximately 0.5 mm from the dentin–enamel junction by a small round carbide bur (H1SEM.205.010 VPE 5, Komet Dental, Lemgo, Germany). The preparation was carried out at a speed of 2000 rpm without water cooling and with low pressure (each bur was used for no more than five lesions). After the pretreatment of the surface, Cention N was applied. In cavities with RGs, a small portion of the mixture was placed first in the RG, and then the rest of it was placed to fill the cavity and restore the tooth form (working time 3 min). As for cavities with no retentive feature, they were bulk‐filled with the filling material. Cention N was light‐cured for 20 s (1200 mW/cm^2^). Afterward, finishing and removing of excess material were carried out using fine and extra‐fine diamond burs (8852.314.014 or 852EF.314.014, Komet, Gebr. Brasseler GmbH & Co. Germany) under water cooling. Polishing was performed using Optrapol (Ivoclar‐Vivadent, Liechtenstein).

RM‐GIC restorations were placed according to the following protocol: after washing and drying, but not desiccating, Dentin Conditioner 20% (GC, Japan) was applied and rinsed. Afterward, an RM–GIC Fuji II LC (GC, Japan) mixture was placed in a single layer in cavities less than 2 mm in depth. As for cavities that are more than 2 mm in depth, the layering technique was used (each layer was light cured for 20 s). Finishing and polishing were performed the same way as in the Cention N groups.

### 2.6 Calibration Procedures for Clinical Evaluation

Two experienced dentists, neither of whom was the clinical operator in this study, were trained as follows: they assessed the periodontal health of 10 class V restorations according to the assessment methods used in this study. However, these restorations were not a part of the restorations performed for this study. At each follow‐up, one of the two assessors evaluated the periodontal health.

### 2.7 Clinical Evaluation

For each follow‐up, a new form was completed. Evaluators were blinded to previous evaluations during the follow‐up sessions. The periodontal evaluation after the placement of the restorations was carried out the same way as that before the placement. BOP, PI, and PD indexes were used for this purpose. Periodontal health was evaluated at baseline, 1 week after restorations' application, and after 3, 6, 9, and 12 months.

### 2.8 Statistical Analysis

Statistical Package for Social Sciences (SPSS) software for Windows V25.0 (RRID:SCR_002865) was used to analyze the data. Data normality was checked using the Shapiro–Wilk test (*p* < 0.001). Comparison tests were performed using the following tests: Friedman's test for intra‐ and intergroup comparisons for plaque accumulation and PD, the Cochran test for intra‐ and intergroup comparisons for gingival bleeding, the Wilcoxon Signed Rank for intra‐ and intergroup comparisons between two groups or two follow‐ups for PD, and the McNemar test for intergroup comparison of proportions of variables between two groups for gingival bleeding. The significance level (*α*) was set to 0.05 or less.

## 3 Results

**Figure 1:**
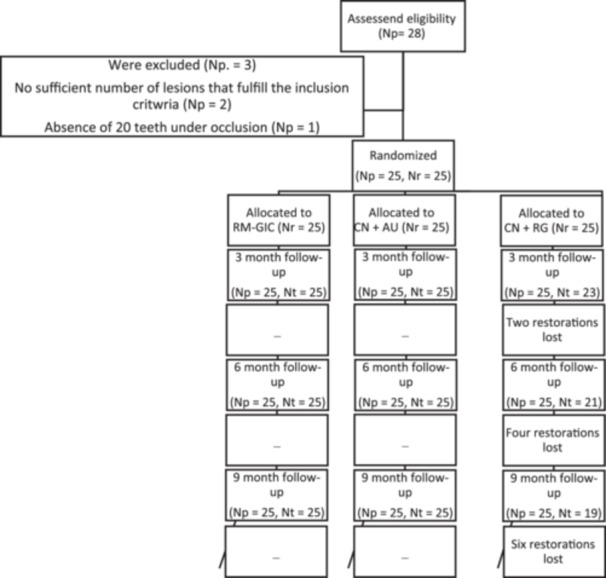
CONSORT flow diagram of the study. Np, number of patients; Nr, number of restorations.

The study sample consisted of 25 patients. A total of 28 patients were examined and their eligibility to participate in the research was determined according to the inclusion and exclusion criteria; 25 of these patients were finally included. The characteristics of the research subjects and NCCLs in this study are shown in Tables 2 and 3. Each patient received three restorations; thus, the total sample size in the study comprised 75 teeth (Figure 1). The response rate was 100% during all follow‐up periods; however, missing data were found for two patients at the 9‐month follow‐up.

**Table 2:** Characteristics of the research subjects.

		*N*	%
Gender	Male	10	40.0
	Female	15	60.0
Parafunctional habits	Rarely/No	11	44.0
	Sometimes	9	36.0
	Often	5	20.0
Smoking	No	17	68.0
	Yes	8	32.0
Age	Mean ± SD	54.32 ± 9.98	

**Table 3:** Characteristics of NCCLs.

	CN + RG	CN + UA	RM‐GIC
	*n* (%)	*n* (%)	*n* (%)
Tooth type			
Canine	2 (8)	2 (8)	2 (8)
Incisor	6 (24)	4 (16)	2 (8)
Molar	4 (16)	4 (16)	4 (16)
Premolar	13 (52)	15 (60)	17 (68)
Tooth arch			
Maxillary	15 (60)	15 (60)	16 (64)
Mandibular	10 (40)	10 (40)	9 (36)

### 3.1 PI

The results of the statistical analysis conducted on the PI data show that there is no significant difference between the groups at each follow‐up (*p* > 0.05) (Supporting Information S1: Table 7S). As for the difference between the follow‐ups for each group, the increase noted for all groups over time was not significant (mean increase = 0.06) (*p* > 0.05) (Supporting Information S1: Table 8S).

### 3.2 PD

As for the PD, there was no significant difference between the groups at all follow‐ups (*p* > 0.05), except for the T3 follow‐up (Supporting Information S1: Table 9S), as the significant difference was between CN + UA and RM‐GIC (*p* = 0.022) (Supporting Information S1: Table 10S).

On comparing the follow‐ups in each group, a significant difference was noted for both CN + AU (*p* = 0.005) and CN + RG (*p* = 0.001) groups (Table 4), with a mean increase of 0.34 between *T*0 and *T*4 for the three groups. To determine if the increase in the mean values of PD was significant after 1 week and after 9 months, the Wilcoxon Signed Ranks Test was conducted. There was a significant difference between the follow‐ups before the intervention on the one hand and after 1 week and 9 months on the other hand for the three groups (*p* > 0.05), except for the comparison between *T*0 and *T*4 in the RM‐GIC group, where the difference was almost significant (*p* = 0.053). Listwise deletion was applied.

**Table 4:** Comparison of probing depth (PD) between different time points for CN + RG, CN + UA, and RM‐GIC.

*T*	CN + RG				CN + UA				RM‐GIC			
	*n*	Mean	SD	Median	*n*	Mean	SD	Median	*N*	Mean	SD	Median
*T*0	17	1.41	0.41	1.33	23	1.48	0.55	1.33	23	1.55	0.49	1.67
*T*1	17	1.98	0.52	2	23	1.90	0.60	2	23	1.90	0.41	2
*T*2	17	1.90	0.48	2	23	1.90	0.47	2	23	1.84	0.37	1.67
*T*3	17	2	0.44	2	23	1.88	0.55	2	23	1.72	0.62	1.67
*T*4	17	1.78	0.33	1.67	23	1.91	0.55	2	23	1.86	0.58	2
*p‐*value^a^	0.001*				0.005*				0.072			

*Note:* T0: before the intervention, T1: baseline (after one week), T2: after 3 months, T3: after 6 months, and T4: after 9 months.

^a^ Friedman Tests.

* Significant at the 0.05 level.

### 3.3 BOP

Regarding BOP, there was no significant difference between the groups in any follow‐up period (*p* > 0.05), as shown in Table 5. However, there was a significant difference in both CN + AU (*p* = 0.027) and RM‐GIC (*p* < 0.001) groups on comparing the follow‐ups.

**Table 5:** Comparison of bleeding on probing (BOP) percentage between different time points for CN + RG, CN + UA, and RM‐GIC and between CN + RG, CN + UA, and RM‐GIC at different time points.

	CN + RG					CN + UA					RM‐GIC					*p‐*value^a^				
	*T*0	*T*1	*T*2	*T*3	*T*4	*T*0	*T*1	*T*2	*T*3	*T*4	*T*0	*T*1	*T*2	*T*3	*T*4	*T*0	*T*1	*T*2	*T*3	*T*4
*N*																				
%																				
Absent	67	60	62	54	47	67	56	65	64	60	68	52	68	68	63	0.939	0.202	0.472	0.705	0.794
	89.3	80	89.9	85.7	92.2	89.3	74.7	86.7	85.3	87	90.7	69.3	90.7	90.7	91.3					
Present	8	15	7	9	4	8	19	10	11	9	7	23	7	7	6					
	10.7	20	10.1	14.3	7.8	10.7	25.3	13.3	14.7	13	9.3	30.7	9.3	9.3	8.7					
*p*‐value^a^	0.234					0.027*					< 0.001*									

*Note:* *T*0: before the intervention, *T*1: baseline (after one week), *T*2: after 3 months, *T*3: after 6 months, and *T*4: after 9 months.

^a^ Cochran Tests.

* Significant at the 0.05 level.

To find out if the increase in the percentage of positive BOP sites was significant after 1 week, the Cochran Test was conducted (Table 6). The test showed that the increase was significant for the CN + AU (*p* = 0.035) and RM‐GIC (*p* = 0.003) groups. However, the BOP sites' percentage decreased to almost the pre‐intervention level during the 3‐, 6‐, and 9‐month follow‐ups for all groups.

**Table 6:** Comparison of bleeding on probing (BOP) percentage between the follow‐ups before the intervention and the baseline for CN + RG, CN + UA, and RM‐GIC.

	Group	Time (*i*) versus Time (*j*)	*p‐*value^a^
BOP	CN + RG	*T*0 versus *T*1	0.210
	CN + UA	*T*0 versus *T*1	0.035*
	RM‐GIC	*T*0 versus *T*1	0.003*

*Note:* *T*0: before the intervention, *T*1: baseline (after one week).

^a^ McNemar Test.

* Significant at the 0.05 level.

## 4 Discussion

There is a strong relationship between the tooth structure and periodontal tissues; still, there are few clinical studies on this topic (Padbury, Eber, and Wang 2003). This randomized‐controlled trial sheds light on an important topic that is usually neglected, by evaluating the periodontal health of teeth restored by two ion‐releasing materials.

The exclusion criteria in the research included pregnancy and lactation, as these variables could affect the periodontal status (Aghazadeh et al. 2019; Laine 2002). Although the relation between bruxism and cervical restoration loss is not conclusive, individuals who reported active severe bruxism habits were also excluded to minimize the risk of restoration loss during the follow‐ups.

The restorations' retention rate for the CN + UA and RM‐GIC groups was 100%, whereas 76% of restorations were present in the CN + RG group after 9 months. However, discussion of these outcomes is beyond the scope of this study.

The statistical analysis for plaque accumulation showed no significant difference between the study groups during any of the follow‐ups. This finding is consistent with those of a previous study comparing RM‐GIC and micro‐filled RC in subgingival restorations (Santos et al. 2007). These findings are also aligned with the results of two studies on NCCLs (Carvalho et al. 2018; Shinohara et al. 2020), which suggest no clinically detectable difference between different restorative materials in the short term.

An increase in the mean PI values was noted during the 3‐month follow‐up and these values remained higher than those before the intervention during both the 6‐ and 9‐month follow‐ups, without a significant difference. Although the presence of restorations is a factor in increasing plaque accumulation and, as a result, the development of periodontal disease, there is not enough evidence to show this relationship (Miller et al. 2003). However, a previous study evaluated the plaque accumulation on anterior RC restorations at a follow‐up of 5–6 years; this retrospective study found that the presence of restorations had a negative effect on the gingival tissue, as their presence led to an increase in plaque accumulation (Peumans et al. 1998). The disagreement between the present study and the previous study may be due to the difference in the type of restorative material used; an in vitro study showed that the surface roughness of Cention N was the lowest compared with 6 other restorative materials, including RC (Kaptan, Oznurhan, and Candan 2023), which may cause less plaque accumulation. The difference in the follow‐up duration could be another reason for disagreement.

This study found no significant difference in PD between the groups during all follow‐up periods. This indicates that the type of restoration does not affect the PD. This result is consistent with that of two previous studies that have also compared the PD between RM‐GIC and RC after the placement of NCCLs restorations (Carvalho et al. 2018; Lucchesi et al. 2007). However, the current study found an increase in PD between the period before the placement of the restorations and after a week; this increase in the means of PD remained almost constant during the follow‐up of 3, 6, and 9 months, and was significant in the CN + RG and CN + UA groups. In contrast, the PD did not increase in two previous studies after the placement of the restorations (Carvalho et al. 2018; Paolantonio et al. 2004). This inconsistency between the two studies and this study might be explained by the difference in the isolation method used, as this study used rubber dam isolation. whereas the two studies used only cotton rolls; the application of the gingival retraction cord and the rubber dam clamps could have had an adverse effect on the gingival tissue in terms of the PD. Unfortunately, so far, no study has evaluated the PD for Class V restorations applied with rubber dam isolation, and thus this conclusion cannot be confirmed or refuted. However, a clinical study has indicated that scaling of pockets less than 4 mm in depth will result in loss of attachment (Cortellini and Tonetti 2011). As both the application of the retentive cord and the scaling procedure lead to physical pressure and trauma on the periodontal tissue, this study may partly explain the loss of attachment occurring in our study. Regarding the placement of the retraction cord, the epithelial attachment withstands forces less than 1 N/mm^2^ and breaks when a force of 2.5 N/mm^2^ is applied, whereas the application of a gingival retraction cord requires an application force of approximately 2.5 N/mm^2^ (Phatale et al. 2010). Additionally, a meta‐analysis reported an increase in PD when applying the gingival retraction cord compared with cordless retraction methods, which may also partly explain the increased PD in our study (Wang et al. 2019). In conclusion, the application of the gingival retraction cord with a rubber dam could explain the loss of attachment. Nonetheless, the average loss value in this study was 0.4 mm; thus, the average PD increased from 1.5 to 1.9 mm, which is still within the normal range of PD.

This study found no significant difference between the study groups during any follow‐up period regarding BOP, indicating no clinically detectable effect of the restoration's material type (CN or RM‐GIC) on the gingival inflammatory response. To the best of our knowledge, there are no studies available on gingival inflammation or gingival health when restoring NCCLs with Cention N; however, one study compared gingival inflammation when restoring pulpotomized primary teeth with Cention N or stainless‐steel crowns and found that the gingival tissue adjacent to teeth restored with Cention N showed a better gingival response (Kaur et al. 2023). These results are in agreement with those of the present study.

However, this study found an increase in the percentage of bleeding sites between the period before the placement of restorations and 1 week after, in the three study groups, with this variance achieving statistical significance in the CN + UA and RM‐GIC groups. Nevertheless, during the 3‐month follow‐up, this percentage decreased to values similar to those before the intervention and remained around these pre‐intervention values during the 6‐ and 9‐month follow‐ups. This can be attributed to the adverse effect of applying the gingival retraction cord and rubber dam clamp on the gingival tissue and the disappearance of this effect in terms of gingival bleeding after a period of less than 3 months. To determine which of the two factors, applying gingival cord or applying a rubber dam, had a greater effect on increasing gingival bleeding at the follow‐up of 1 week, the results of this study were compared with those of a study that applied only a gingival cord without the rubber dam for the purpose of taking an impression. In the latter study, the number of bleeding sites did not increase after a day or 10 days, indicating that the rubber dam's clamp could have played a greater role in increasing bleeding sites at the 1‐week follow‐up in the present study (Sarmento et al. 2014). Another study also reported the negative effect of applying a rubber dam's clamp on gingival recession, which may be a result of gingival trauma and inflammation during the period after the restorative procedures (Favetti et al. 2021).

Of particular interest is the fact that the BOP was not affected by the presence of a restoration, despite a noted increase in plaque accumulation after the restorations' application. This could be attributable to the effect of the type of restoration applied on the compensation of the plaque. Evidence from a clinical investigation demonstrated a reduction in the pathogenic count within the bacterial plaque in teeth restored with RM‐GIC (Santos et al. 2007). This reduction may be due to the effect of the release of different ions from RM‐GIC, as the released fluoride interferes with the process of initial adhesion of bacteria to the surface of the restoration and inhibits the metabolism and growth of bacteria (van Dijken, Persson, and Sjöström 1991; van Dijken and Sjöström 1991). As for the two Cention N groups, Cention N incorporates silanized fillers, which are highly reactive, particularly in acidic environments (Tiskaya et al. 2019); this could have a similar antibacterial effect as RM‐GIC. Additionally, the effect of this material on Streptococcus mutans and the increase in the pH of the bacterial plaque may have an impact on the composition of the bacterial plaque and its ability to cause gingivitis (Aparajitha et al. 2021; Daabash et al. 2023; Feiz et al. 2022; Wiriyasatiankun, Sakoolnamarka, and Thanyasrisung 2022). Apparently, there could have been an important effect of the ion‐release properties of the material on plaque composition. What further supports this point are the results of two microbiological investigations conducted by Paolantonio et al. and Santos et al. that found an adverse impact of RC on the amount and count of subgingival bacterial plaque compared with GIC and amalgam (Paolantonio et al. 2004; Santos et al. 2007). Additionally, one cross‐sectional study on teeth restored with traditional nonion‐releasing RC found a significant difference between unrestored and restored NCCLs (Gurgel et al. 2016). Nevertheless, two clinical studies contradict this conclusion. The first one compared RC and RM‐GIC when repairing NCCLs and noted no significant difference between the two groups at the 3‐ and 6‐month follow‐ups (Carvalho et al. 2018). The second one is the clinical aspect of the study by Santos, V.R., et al, which found that there was no significant difference between RC and RM‐GIC restorations accompanied by a coronally positioned flap procedure in terms of BOP at a follow‐up of 6 months (Santos et al. 2007). This contradiction between these findings and those in the present study could be due to the small sample size in both studies (18 patients per study) and the strict oral care instructions followed when performing flaps in the second study.

As the results of the study show, Cention N and RM‐GIC restorations are treatment options that have a gentle effect on the gingival tissue when restoring NCCLs. However, treatment of shallow NCCLs that do not involve interdental bone loss with connective graft should be considered, as this option yields a better gingival response than restorative treatment after 3 months with regard to periodontal variables in the present study (Leybovich et al. 2014).

This study did not evaluate the subgingival microbial biofilm; thus, future studies are needed to evaluate the effect of different ion‐releasing materials on gingival health in comparison with nonion‐releasing materials, especially in the long term. Some gingival‐related parameters were not evaluated in this study, such as the width of keratinized tissue and the clinical attachment level. The evaluation of these variables and the subgingival extension of the restorations in future studies will provide a more holistic picture of the effect of Cention N and other materials on gingival health.

## 5 Conclusions

Within the limitations of this study, it can be concluded that: The clinical periodontal‐related performance of Cention N is comparable to that of RM‐GIC. The presence of these ion‐releasing restorations had no clinically detectable effect on gingival inflammation. Nonetheless, it is noteworthy that the application of both the retraction cord and the rubber dam clamp may play a role in precipitating attachment loss.

## Author Contributions

**Khattab Mustafa:** study conception and design, visualization, methodology, project administration, analysis and interpretation of results, resources, original draft preparation, writing–review and editing. **Ghaith Alfakhry:** writing–review and editing. **Hussam Milly:** supervision, visualization, writing–review and editing.

## Ethics Statement

Ethical approval was obtained from Damascus University no./2777/2021. Written informed consent was obtained from all participants before starting the restorative procedures.

## Conflicts of Interest

The authors declare no conflicts of interest.

## Supporting information

Supporting information.

## Data Availability

The data that support the findings of this study are available from the corresponding author upon reasonable request.

## References

[cre270025-bib-0001] Aghazadeh, Z. , A. Behroozian , H. Najaf , and M. Faramarzi . 2019. “Comparison of Gingival and Dental Indices in Lactating and Non‐Lactating Mothers During First 6 Month After Delivery.” Pesquisa Brasileira em Odontopediatria e Clínica Integrada 19: 1–11.

[cre270025-bib-0002] Aparajitha, R. , P. Selvan , A. Ahamed , S. Bhavani , and V. Nagarajan . 2021. “Comparative Evaluation of Long‐Term Fluoride Release and Antibacterial Activity of an Alkasite, Nanoionomer, and Glass Ionomer Restorative Material—An In Vitro Study.” Journal of Conservative Dentistry 24, no. 5: 485–490. 10.4103/jcd.jcd_336_21. 35399765 PMC8989179

[cre270025-bib-0003] Bezerra, I. M. , A. C. M. Brito , S. A. de Sousa , B. M. Santiago , Y. W. Cavalcanti , and L. F. D. de Almeida . 2020. “Glass Ionomer Cements Compared With Composite Resin in Restoration of Noncarious Cervical Lesions: A Systematic Review and Meta‐Analysis.” Heliyon 6, no. 5: e03969. 10.1016/j.heliyon.2020.e03969. 32462087 PMC7243139

[cre270025-bib-0004] Carvalho, R. D. , C. Nogueira , A. Silva , et al. 2018. “Periodontal Evaluation in Noncarious Cervical Lesions Restored With Resin‐Modified Glass‐Ionomer Cement and Resin Composite: A Randomised Controlled Study.” Oral Health & Preventive Dentistry 16, no. 2: 131–136. 10.3290/j.ohpd.a40295. 29736491

[cre270025-bib-0005] Cortellini, P. , and M. S. Tonetti . 2011. “Clinical and Radiographic Outcomes of the Modified Minimally Invasive Surgical Technique With and Without Regenerative Materials: A Randomized‐Controlled Trial in Intra‐Bony Defects: Modified Minimally Invasive Surgery and Intra‐Bony Defects.” Journal of Clinical Periodontology 38: 365–373. 10.1111/j.1600-051X.2011.01705.x. 21303402

[cre270025-bib-0006] Daabash, R. , M. Q. Alqahtani , R. B. Price , A. Alshabib , A. Niazy , and M. M. Alshaafi . 2023. “Surface Properties and Streptococcus Mutans Biofilm Adhesion of Ion‐Releasing Resin‐Based Composite Materials.” Journal of Dentistry 134: 104549. 10.1016/j.jdent.2023.104549. 37196686

[cre270025-bib-0007] van Dijken, J. , S. Persson , and S. Sjöström . 1991. “Presence of Streptococcus Mutans and Lactobacilli in Saliva and on Enamel, Glass Ionomer Cement, and Composite Resin Surfaces.” European Journal of Oral Sciences 99, no. 1: 13–19. 10.1111/j.1600-0722.1991.tb01017.x. 2047748

[cre270025-bib-0008] van Dijken, J. W. V. , and S. Sjöström . 1991. “The Effect of Glass Ionomer Cement and Composite Resin Fillings on Marginal Gingiva.” Journal of Clinical Periodontology 18, no. 3: 200–203. 10.1111/j.1600-051x.1991.tb01134.x. 1829463

[cre270025-bib-0009] Favetti, M. , A. F. Montagner , S. T. Fontes , et al. 2021. “Effects of Cervical Restorations on the Periodontal Tissues: 5‐year Follow‐Up Results of a Randomized Clinical Trial.” Journal of Dentistry 106: 103571. 10.1016/j.jdent.2020.103571. 33385534

[cre270025-bib-0010] Feiz, A. , M. Nicoo , A. Parastesh , N. Jafari , and D. Sarfaraz . 2022. “Comparison of Antibacterial Activity and Fluoride Release in Tooth‐Colored Restorative Materials: Resin‐Modified Glass Ionomer, Zirconomer, Giomer, and Cention N.” Dental Research Journal 19, no. 1: 104. 10.4103/1735-3327.363534. 36605145 PMC9807936

[cre270025-bib-0011] Goodacre, C. J. , W. Eugene Roberts , and C. A. Munoz . 2023. “Noncarious Cervical Lesions: Morphology and Progression, Prevalence, Etiology, Pathophysiology, and Clinical Guidelines for Restoration.” Journal of Prosthodontics 32, no. 2: e1–e18. 10.1111/jopr.13585. 35920595

[cre270025-bib-0012] Gurgel, B. C. , N. G. Solera , R. F. Peixoto , A. O. Assis , P. D. Calderon , and M. C. Medeiros . 2016. “Evaluation of the Periodontal Conditions of Teeth With Restored and Non‐Restored Non‐Carious Cervical Lesions.” Quintessence international (Berlin, Germany: 1985) 47, no. 10: 825–831. 10.3290/j.qi.a36885. 27669720

[cre270025-bib-0013] Kaptan, A. , F. Oznurhan , and M. Candan . 2023. “In Vitro Comparison of Surface Roughness, Flexural, and Microtensile Strength of Various Glass‐Ionomer‐Based Materials and a New Alkasite Restorative Material.” Polymers 15, no. 3: 650. 10.3390/polym15030650. 36771950 PMC9920171

[cre270025-bib-0014] Kaur, K. , B. Suneja , S. Jodhka , et al. 2023. “Comparison Between Restorative Materials for Pulpotomised Deciduous Molars: A Randomized Clinical Study.” Children 10, no. 2: 284. 10.3390/children10020284. 36832414 PMC9955046

[cre270025-bib-0015] Laine, M. A. 2002. “Effect of Pregnancy on Periodontal and Dental Health.” Acta Odontologica Scandinavica 60, no. 5: 257–264. 10.1080/00016350260248210. 12418714

[cre270025-bib-0016] Leybovich, M. , N. F. Bissada , S. Teich , C. A. Demko , and P. A. Ricchetti . 2014. “Treatment of Noncarious Cervical Lesions By a Subepithelial Connective Tissue Graft Versus a Composite Resin Restoration.” International Journal of Periodontics & Restorative Dentistry 34, no. 5: 649–654. 10.11607/prd.2033. 25171035

[cre270025-bib-0017] Loguercio, A. D. , I. V. Luque‐Martinez , S. Fuentes , A. Reis , and M. A. Muñoz . 2018. “Effect of Dentin Roughness on the Adhesive Performance in Non‐Carious Cervical Lesions: A Double‐Blind Randomized Clinical Trial.” Journal of Dentistry 69: 60–69. 10.1016/j.jdent.2017.09.011. 28962842

[cre270025-bib-0018] Lucchesi, J. A. , V. R. Santos , C. M. Amaral , D. C. Peruzzo , and P. M. Duarte . 2007. “Coronally Positioned Flap for Treatment of Restored Root Surfaces: A 6‐month Clinical Evaluation.” Journal of Periodontology 78, no. 4: 615–623. 10.1902/jop.2007.060380. 17397307

[cre270025-bib-0019] Miller, N. , J. Penaud , P. Ambrosini , C. Bisson‐Boutelliez , and S. Briançon . 2003. “Analysis of Etiologic Factors and Periodontal Conditions Involved With 309 Abfractions.” Journal of Clinical Periodontology 30, no. 9: 828–832. 10.1034/j.1600-051x.2003.00378.x. 12956659

[cre270025-bib-0020] Naik, V. , C. Jacob , and D. Nainar . 2016. “Assessment of Non‐Carious Root Surface Defects in Areas of Gingival Recession: A Descriptive Study.” Journal of Clinical and Experimental Dentistry 8, no. 4: e397–e402. 10.4317/jced.52831. 27703607 PMC5045686

[cre270025-bib-0021] Padbury, A. Jr. , R. Eber , and H. L. Wang . 2003. “Interactions Between the Gingiva and the Margin of Restorations.” Journal of Clinical Periodontology 30, no. 5: 379–385. 10.1034/j.1600-051x.2003.01277.x. 12716328

[cre270025-bib-0022] Paolantonio, M. , S. D'Ercole , G. Perinetti , et al. 2004. “Clinical and Microbiological Effects of Different Restorative Materials on the Periodontal Tissues Adjacent to Subgingival Class V Restorations.” Journal of Clinical Periodontology 31, no. 3: 200–207. 10.1111/j.0303-6979.2004.00472.x. 15016024

[cre270025-bib-0023] Peumans, M. , B. Van Meerbeek , P. Lambrechts , G. Vanherle , and M. Quirynen . 1998. “The Influence of Direct Composite Additions for the Correction of Tooth Form and/or Position on Periodontal Health. A Retrospective Study.” Journal of Periodontology 69, no. 4: 422–427. 10.1902/jop.1998.69.4.422. 9609371

[cre270025-bib-0024] Phatale, S. , P. Marawar , G. Byakod , S. Lagdive , and J. Kalburge . 2010. “Effect of Retraction Materials on Gingival Health: A Histopathological Study.” Journal of Indian Society of Periodontology 14, no. 1: 35–39. 10.4103/0972-124X.65436. 20922077 PMC2933527

[cre270025-bib-0025] Ruengrungsom, C. , M. F. Burrow , P. Parashos , and J. E. A. Palamara . 2020. “Evaluation of F, Ca, and P Release and Microhardness of Eleven Ion‐Leaching Restorative Materials and the Recharge Efficacy Using a New Ca/P Containing Fluoride Varnish.” Journal of Dentistry 102: 103474. 10.1016/j.jdent.2020.103474. 32941973

[cre270025-bib-0026] Santos, V. R. , J. A. Lucchesi , S. C. Cortelli , C. M. Amaral , M. Feres , and P. M. Duarte . 2007. “Effects of Glass Ionomer and Microfilled Composite Subgingival Restorations on Periodontal Tissue and Subgingival Biofilm: A 6‐month Evaluation.” Journal of Periodontology 78, no. 8: 1522–1528. 10.1902/jop.2007.070032. 17668971

[cre270025-bib-0027] Sarmento, H. R. , F. R. M. Leite , R. V. F. Dantas , F. A. Ogliari , F. F. Demarco , and F. Faot . 2014. “A Double‐Blind Randomised Clinical Trial of Two Techniques for Gingival Displacement.” Journal of Oral Rehabilitation 41, no. 4: 306–313. 10.1111/joor.12142. 24446590

[cre270025-bib-0028] Sarmento, H. R. , F. R. M. Leite , R. V. F. Dantas , F. A. Ogliari , F. F. Demarco , and F. Faot . 2014. “A Double‐Blind Randomised Clinical Trial of Two Techniques for Gingival Displacement.” Journal of Oral Rehabilitation 41, no. 5: 306–313. 10.1111/joor.12142. 24446590

[cre270025-bib-0029] Schulz, K. F. , D. G. Altman , and D. Moher . 2010. “Consort 2010 Statement: Updated Guidelines for Reporting Parallel Group Randomised Trials.” Bmj 340: c332. 10.1136/bmj.c332. 20332509 PMC2844940

[cre270025-bib-0030] Silness, J. , and H. Löe . 1964. “Periodontal Disease in Pregnancy II. Correlation Between Oral Hygiene and Periodontal Condition.” Acta Odontologica Scandinavica 22: 121–135. 10.3109/00016356408993968. 14158464

[cre270025-bib-0031] Teixeira, D. N. R. , R. Z. Thomas , P. V. Soares , M. S. Cune , M. M. M. Gresnigt , and D. E. Slot . 2020. “Prevalence of Noncarious Cervical Lesions Among Adults: A Systematic Review.” Journal of Dentistry 95: 103285. 10.1016/j.jdent.2020.103285. 32006668

[cre270025-bib-0032] Tiskaya, M. , N. A. Al‐Eesa , F. S. L. Wong , and R. G. Hill . 2019. “Characterization of the Bioactivity of Two Commercial Composites.” Dental Materials 35, no. 12: 1757–1768. 31699444 10.1016/j.dental.2019.10.004

[cre270025-bib-0033] Wang, Y. , F. Fan , X. Li , et al. 2019. “Influence of Gingival Retraction Paste Versus Cord on Periodontal Health: A Systematic Review and Meta‐Analysis.” Quintessence international (Berlin, Germany: 1985) 50, no. 3: 234–244. 10.3290/j.qi.a41976. 30773575

[cre270025-bib-0034] Wiriyasatiankun, P. , R. Sakoolnamarka , and P. Thanyasrisung . 2022. “The Impact of an Alkasite Restorative Material on the pH of Streptococcus Mutans Biofilm and Dentin Remineralization: An in Vitro Study.” BMC Oral Health 22, no. 1: 334. 10.1186/s12903-022-02354-4. 35941628 PMC9361645

