# Supplementary material for: Periodontal Evaluation for a New Alkasite Restorative Material in Noncarious Cervical Lesions: A Randomized‐Controlled Clinical Trial
Source: Clin Exp Dent Res. 2024 Oct 13;10(6):e70025. doi: 10.1002/cre2.70025 (PMC11471885; doi:10.1002/cre2.70025)
Supplement: Supplementary file 1 — Supporting information. [file CRE2-10-e70025-s001.docx]

**Supplementary Tables:**

**Table 7S Comparison of plaque index (PI), between CN + RG and CN +UA (test groups) and RM-GIC (control group) at different time points.**

| T | CN + RG | | | | CN + UA | | | | RM-GIC | | | | P-value^a^ |
| --- | --- | --- | --- | --- | --- | --- | --- | --- | --- | --- | --- | --- | --- |
|  | n | Mean | SD | Median | n | Mean | SD | Median | N | Mean | SD | Median |  |
| T0 | 25 | 0.32 | 0.69 | 0 | 25 | 0.36 | 0.70 | 0 | 25 | 0.32 | 0.63 | 0 | 0.913 |
| T1 | 25 | 0.36 | 0.57 | 0 | 25 | 0.4 | 0.58 | 0 | 25 | 0.44 | 0.77 | 0 | 0.852 |
| T2 | 23 | 0.57 | 0.66 | 0 | 23 | 0.57 | 0.59 | 1 | 23 | 0.52 | 0.59 | 0 | 0.905 |
| T3 | 20 | 0.55 | 0.60 | 0.5 | 20 | 0.50 | 0.61 | 0 | 20 | 0.55 | 0.51 | 1 | 0.846 |
| T4 | 17 | 0.35 | 0.61 | 0 | 17 | 0.35 | 0.49 | 0 | 17 | 0.24 | 0.44 | 0 | 0.687 |
| a. Friedman Test.  T0: before the intervention, T1: baseline (after one week), T2: after 3 months, T3: after 6 months, T4: after 9 months. | | | | | | | | | | | | | |

**Table 8S Comparison of plaque index (PI), between different time points for CN + RG, CN +UA, and RM-GIC.**

| T | CN + RG | | | | CN + UA | | | | RM-GIC | | | |
| --- | --- | --- | --- | --- | --- | --- | --- | --- | --- | --- | --- | --- |
|  | N | Mean | SD | Median | n | Mean | SD | Median | n | Mean | SD | Median |
| T0 | 17 | 0.29 | 0.69 | 0 | 23 | 0.32 | 0.65 | 0 | 23 | 0.26 | 0.54 | 0 |
| T1 | 17 | 0.24 | 0.44 | 0 | 23 | 0.32 | 0.48 | 0 | 23 | 0.30 | 0.56 | 0 |
| T2 | 17 | 0.53 | 0.72 | 0 | 23 | 0.50 | 0.60 | 0 | 23 | 0.48 | 0.59 | 0 |
| T3 | 17 | 0.41 | 0.51 | 0 | 23 | 0.50 | 0.60 | 0 | 23 | 0.52 | 0.51 | 0 |
| T4 | 17 | 0.35 | 0.61 | 0 | 23 | 0.41 | 0.50 | 0 | 23 | 0.30 | 0.47 | 0 |
| P-value^a^ | 0.554 | | | | 0.506 | | | | 0.226 | | | |
| a. Friedman Tests.  T0: before the intervention, T1: baseline (after one week), T2: after 3 months, T3: after 6 months, T4: after 9 months. | | | | | | | | | | | | |

**Table 9S Comparison of probing depth (PD), between CN + RG and CN +UA (test groups) and RM-GIC (control group) at different time points.**

| T | CN + RG | | | | CN + UA | | | | RM-GIC | | | | P-value^a^ |
| --- | --- | --- | --- | --- | --- | --- | --- | --- | --- | --- | --- | --- | --- |
|  | N | Mean | SD | Median | N | Mean | SD | Median | N | Mean | SD | Median |  |
| T0 | 25 | 1.47 | 0.40 | 1.33 | 25 | 1.57 | 0.68 | 1.33 | 25 | 1.59 | 0.48 | 1.67 | 0.201 |
| T1 | 25 | 1.93 | 0.54 | 2.00 | 25 | 1.89 | 0.58 | 2.00 | 25 | 1.92 | 0.40 | 2.00 | 0.948 |
| T2 | 23 | 1.81 | 0.48 | 1.67 | 23 | 1.87 | 0.45 | 1.67 | 23 | 1.84 | 0.37 | 1.67 | 0.902 |
| T3 | 21 | 1.94 | 0.60 | 2.00 | 21 | 1.94 | 0.66 | 2.00 | 21 | 1.75 | 0.67 | 1.67 | 0.027* |
| T4 | 17 | 1.78 | 0.33 | 1.67 | 17 | 1.84 | 0.52 | 1.67 | 17 | 1.78 | 0.56 | 1.67 | 0.867 |
| a. Friedman Test.  * significant at the 0.05 level.  T0: before the intervention, T1: baseline (after one week), T2: after 3 months, T3: after 6 months, T4: after 9 months. | | | | | | | | | | | | | |

**Table 10S Comparison of probing depth (PD), between every two study groups at six-month follow-up.**

|  | | Group (i) vs Group (j) | P-value^a^ |
| --- | --- | --- | --- |
| PD | T3 | CN + RG VS CN + UA | 0.754 |
|  |  | CN + RG VS RM-GIC | 0.075 |
|  |  | CN + UA VS RM-GIC | 0.022* |
| a. Wilcoxon Signed Ranks Test.  * significant at the 0.05 level.  T3: after 6 months. | | | |
